# Phylogenetic Analysis Indicates That Evasin-Like Proteins of Ixodid Ticks Fall Into Three Distinct Classes

**DOI:** 10.3389/fcimb.2021.769542

**Published:** 2021-10-22

**Authors:** Shoumo Bhattacharya, Patricia Anne Nuttall

**Affiliations:** ^1^ Division of Cardiovascular Medicine, Radcliffe Department of Medicine, Wellcome Centre for Human Genetics, University of Oxford, Oxford, United Kingdom; ^2^ National Institute for Health Research (NIHR) Oxford Biomedical Research Centre, John Radcliffe Hospital, Oxford University Hospitals National Health Service (NHS) Foundation Trust, Oxford, United Kingdom; ^3^ Department of Zoology, University of Oxford, Oxford, United Kingdom

**Keywords:** chemokine binding protein, tick, transcriptome, salivary glands, phylogenetic analyses, evasin

## Abstract

Chemokines are structurally related proteins that activate leucocyte migration in response to injury or infection. Tick saliva contains chemokine-binding proteins or evasins which likely neutralize host chemokine function and inflammation. Biochemical characterisation of 50 evasins from *Ixodes*, *Amblyomma* and *Rhipicephalus* shows that they fall into two functional classes, A and B, with exclusive binding to either CC- or CXC- chemokines, respectively. Class A evasins, EVA1 and EVA4 have a four-disulfide-bonded core, whereas the class B evasin EVA3 has a three-disulfide-bonded “knottin” structure. All 29 class B evasins have six cysteine residues conserved with EVA3, arrangement of which defines a Cys6-motif. Nineteen of 21 class A evasins have eight cysteine residues conserved with EVA1/EVA4, the arrangement of which defines a Cys8-motif. Two class A evasins from *Ixodes* (IRI01, IHO01) have less than eight cysteines. Many evasin-like proteins have been identified in tick salivary transcriptomes, but their phylogenetic relationship with respect to biochemically characterized evasins is not clear. Here, using BLAST searches of tick transcriptomes with biochemically characterized evasins, we identify 292 class A and 157 class B evasins and evasin-like proteins from Prostriate (*Ixodes*), and Metastriate (*Amblyomma, Dermacentor, Hyalomma*, *Rhipicephalus*) ticks. Phylogenetic analysis shows that class A evasins/evasin-like proteins segregate into two classes, A1 and A2. Class A1 members are exclusive to Metastriate ticks and typically have a Cys8-motif and include EVA1 and EVA4. Class A2 members are exclusive to Prostriate ticks, lack the Cys8-motif, and include IHO01 and IRI01. Class B evasins/evasin-like proteins are present in both Prostriate and Metastriate lineages, typically have a Cys6-motif, and include EVA3. Most evasins/evasin-like proteins in Metastriate ticks belong to class A1, whereas in Prostriate species they are predominantly class B. In keeping with this, the majority of biochemically characterized Metastriate evasins bind CC-chemokines, whereas the majority of Prostriate evasins bind CXC-chemokines. While the origin of the structurally dissimilar classes A1 and A2 is yet unresolved, these results suggest that class B evasin-like proteins arose before the divergence of Prostriate and Metastriate lineages and likely functioned to neutralize CXC-chemokines and support blood feeding.

## Introduction

Chemokines are a family of structurally related proteins that have prominent roles in driving leucocyte recruitment - a process that plays a key role not only in the inflammatory response to injury or infection (predominantly by neutrophils and monocyte/macrophages), but also in immune system homeostasis mediated by trafficking of lymphocytes and dendritic cells (Zlotnik and Yoshie, 2012). The >45 mammalian chemokines are grouped according to the spacing of their N-terminal cysteine residues into CC, CXC, CX3C and XC classes. They bind to a family of 19 G-protein coupled receptors (GPCRs) that are expressed on leucocytes. Activation of GPCR-signalling leads to directed migration or chemotaxis of leucocytes to the site of chemokine expression, for instance, to the site of infection or injury, where they form the first line of defence. The chemokine network is highly redundant with multiple chemokines expressed at the site of injury, many chemokines binding multiple receptor subtypes, and multiple receptor subtypes typically expressed on a single leucocyte subclass (Mantovani, 2018; Bhattacharya and Kawamura, 2020). This redundancy is thought to result in a robust output that is resistant to genetic or environmental variation (Mantovani, 2018).

The site of a tick bite is characterized by early expression of several CC and CXC-class chemokines (Glatz et al., 2017). Following *Ixodes ricinus* bite in humans, elevated levels of CCL2, CCL3, CCL4, CXCL1 and CXCL8 are observed, and there is early accumulation of macrophages and dendritic cells (Glatz et al., 2017). Following *Ixodes scapularis* bites in mice, there is local expression of CCL2, CCL7, CXCL2, CXCL5, but not CXCL3, and accumulation of neutrophils, eosinophils, lymphocytes and histiocytes (i.e., tissue monocyte/macrophages) (Krause et al., 2009; Heinze et al., 2012). However, the area immediately adjacent to the tick hypostome is paucicellular - i.e. minimally inflamed, and this is thought to be due to anti-inflammatory effects of tick salivary proteins (Krause et al., 2009). Reinfestation is associated with infiltration of the paucicellular zone with neutrophils, eosinophils, lymphocytes and histiocytes, factors that cause premature tick detachment (Krause et al., 2009). Indeed, basophils appear to have a critical role in acquired resistance to tick reinfestation (Wada et al., 2010).

The nature of the biological arsenal with which ticks may defend against redundant chemokine-induced inflammation has become clearer following the initial report of chemokine-binding proteins in tick saliva (Hajnická et al., 2001). Ticks produce salivary chemokine-binding proteins that bind and neutralize multiple chemokines – which would be expected to minimize local inflammation and allow prolonged blood feeding. Three proteins - evasins 1, 3 and 4 (EVA1, EVA3, EVA4) - were cloned from the salivary gland of the dog tick *Rhipicephalus sanguineus* (Frauenschuh et al., 2007; Deruaz et al., 2008). EVA1 and EVA4 specifically bind CC chemokines, whereas EVA3 specifically binds CXC chemokines. Structural characterization of the CC-binding evasins, EVA1 and EVA4, indicates a four-disulfide-bonded core structure (Dias et al., 2009; Denisov et al., 2020). Characterization of the CXC-chemokine-binding evasin EVA3 indicates that it forms a three disulfide-bonded “knottin” motif (Denisov et al., 2019; Lee et al., 2019). While “knottin” structures are reported in multiple organisms, evasins appear to be unique to ticks as sequence homologs have not been reported for any other organisms to date.

In addition to inflammation inhibitors, tick saliva is known to contain inhibitors of blood clotting, complement activation, pain, and itch – many of which have potential therapeutic and vaccine development applications (Simo et al., 2017; Aounallah et al., 2020). The advent of cost-effective RNA sequencing has fuelled a veritable explosion of sequence data from the salivary transcriptomes of a number of tick species (Ribeiro and Mans, 2020). This serves as a valuable resource for identifying novel modulators of human biology that have appeared in ticks through natural selection.

By searching tick salivary gland transcriptome sequence databases for homologs of published evasins, many evasin-like proteins have been identified in diverse tick genera. By selecting candidates for biochemical characterization of expressed proteins, we and others have identified a total of 47 further chemokine binding evasins to date (Deruaz et al., 2008; Déruaz et al., 2013; Hayward et al., 2017; Singh et al., 2017; Alenazi et al., 2018; Eaton et al., 2018; Lee et al., 2019). Notably, each cloned evasin binds multiple chemokines from either the CC- or the CXC class, providing an attractive mechanism to overcome the redundancy of the chemokine network. The 29 known CXC-chemokine-binding evasins have a six cysteine residue ‘knottin’ motif conserved with EVA3 with the spacing C-x(3)-C-x(6,10)-C-x(3,6)-C-x(1)-C-x(10,11)-C (Lee et al., 2019), and referred to here as the Cys6-motif. Of the 21 known CC-chemokine-binding evasins, 19 have eight cysteine residues conserved with EVA1/EVA4, with the spacing C-x(14,17) -C-x(3)-C-x(11,16)-C-x(17,20)-C-x(4)-C-x(4,5)-C-x(8)-C), and referred to here as the Cys8-motif (Deruaz et al., 2008; Déruaz et al., 2013; Hayward et al., 2017; Singh et al., 2017; Alenazi et al., 2018; Eaton et al., 2018). However, two CC-chemokine-binding evasins - from *Ixodes* species (IRI01 and IHO01) - have less than eight cysteine residues (Hayward et al., 2017), indicating that a four-disulfide-bonded core cannot be formed.

The 50 validated evasins have been distinguished into two classes, class A evasins (CC-chemokine binders) and class B evasins (CXC-chemokine binders), that each share structural and conserved sequence features although their sequence identity may be <30% (Bhusal et al., 2020). Phylogenetic sequence relationships of these biochemically characterized evasins to the many evasin-like proteins annotated in tick transcriptome databases are unclear. Here, using BLAST searches of tick transcriptomes supported by the 50 biochemically characterized evasins (defined here as possessing a chemokine-binding function), followed by sequence-based phylogenetic tree construction, we identify evasin-like proteins (defined here as ones not yet tested for a chemokine-binding function) and show they fall into three distinct classes.

## Materials and Methods

### Databases

The transcriptome shotgun assembly database (tsa_nr) and the non-redundant protein database (nr) was downloaded from NCBI (May 2021).

### BLAST Search

Databases were searched using command-line BLAST+ blastp (BLAST/2.10.1-Linux_x86_64) on the Oxford BMRC cluster with each of the 21 biochemically characterized class A and 29 biochemically characterized class B evasins (**Supplementary Tables S1**, **S2**, respectively), using default values for blastp and restricting the search to ticks, to retrieve NCBI accession numbers, genus, and species. Distinct sequences with e-value <0.0001 were used for subsequent analysis.

### Identification of Mature Protein Sequence

Subsequent steps were performed with customized R scripts. NCBI accession numbers identified by blastp were used to retrieve the corresponding protein sequences using “entrez_fetch” (package rentrez). The first methionine residue in each sequence was used to identify the protein start site. A local installation of the program SignalP 4.1 (downloaded from https://services.healthtech.dtu.dk) was used to identify the signal peptide (Bendtsen et al., 2004). The start of the mature peptide was identified using the parameter posYmax. Sequences lacking a signal peptide and sequences containing stop codons in the predicted mature protein sequence were filtered out. Proteins identified are shown in **Supplementary Table S1** (class A evasins/evasin-like proteins) and **Supplementary Table S2** (class B evasins/evasin-like proteins).

### Phylogenetic Analysis

Mature protein sequences identified above were characterized for cysteine counts and published evasin cysteine motifs, and information files were generated for the heatmap (**Supplementary Tables S1**, **S2**). Mature protein sequences were aligned using “ClustalW” with the Gonnet substitution matrix (Gonnet et al., 1992), accessed through the package “msa” (Bodenhofer et al., 2015). Aligned sequences were used to construct a matrix of pairwise distances using the function “dist.alignment” [package “seqinr” (Charif and Lobry, 2007)]. Neighbour-joining trees were constructed using the functions “nj” and “boot.phylo” (in package “ape” (Paradis, 2012) with default parameters i.e. 100 bootstrap replicates), and then rooted at the midpoint using the function “midpoint.root” in the package “phytools” (Revell, 2012). The phylogenetic trees were plotted using the package “ggtree” (Yu et al., 2016), and information files generated above added to create the heatmaps. Chemokine binding profiles and binding motifs for evasins were based on published data (Deruaz et al., 2008; Déruaz et al., 2013; Hayward et al., 2017; Singh et al., 2017; Alenazi et al., 2018; Eaton et al., 2018; Lee et al., 2019).

### Software Package Versions

R version 4.1.1 (2021-08-10), platform: x86_64-apple-darwin17.0 (64-bit), running under: macOS Big Sur 11.6, ape_5.5, phytools_0.7-80, ggtree_3.0.4, treeio_1.16.2, tidyverse_1.3.1, ggplot2_3.3.5, BiocGenerics_0.36.0 rentrez_1.2.3, Biostrings_2.60.2, seqinr_4.2-8, msa_1.24.0, taxize_0.9.99, SignalP 4.1 (Bendtsen et al., 2004).

## Results

Sequences of the 50 biochemically characterized evasins (21 in class A and 29 in class B) were used to screen the BLAST databases, which consisted of 42586 argasid, 204166 Metastriate ixodid and 170717 Prostriate ixodid tick sequences (**Supplementary Figure S1A**). Using a BLAST e-value threshold of p< 0.0001, we identified 271 class A and 128 class B evasin-like proteins from Prostriate (*Ixodes*) and Metastriate (*Amblyomma, Dermacentor, Hyalomma*, *Rhipicephalu*s) ixodid ticks (**Supplementary Tables S1**, **S2**). No evasin-like proteins were identified for argasid ticks. A possible reason is that our search criteria are too stringent to identify very distantly related sequences.

**Figure 1:**
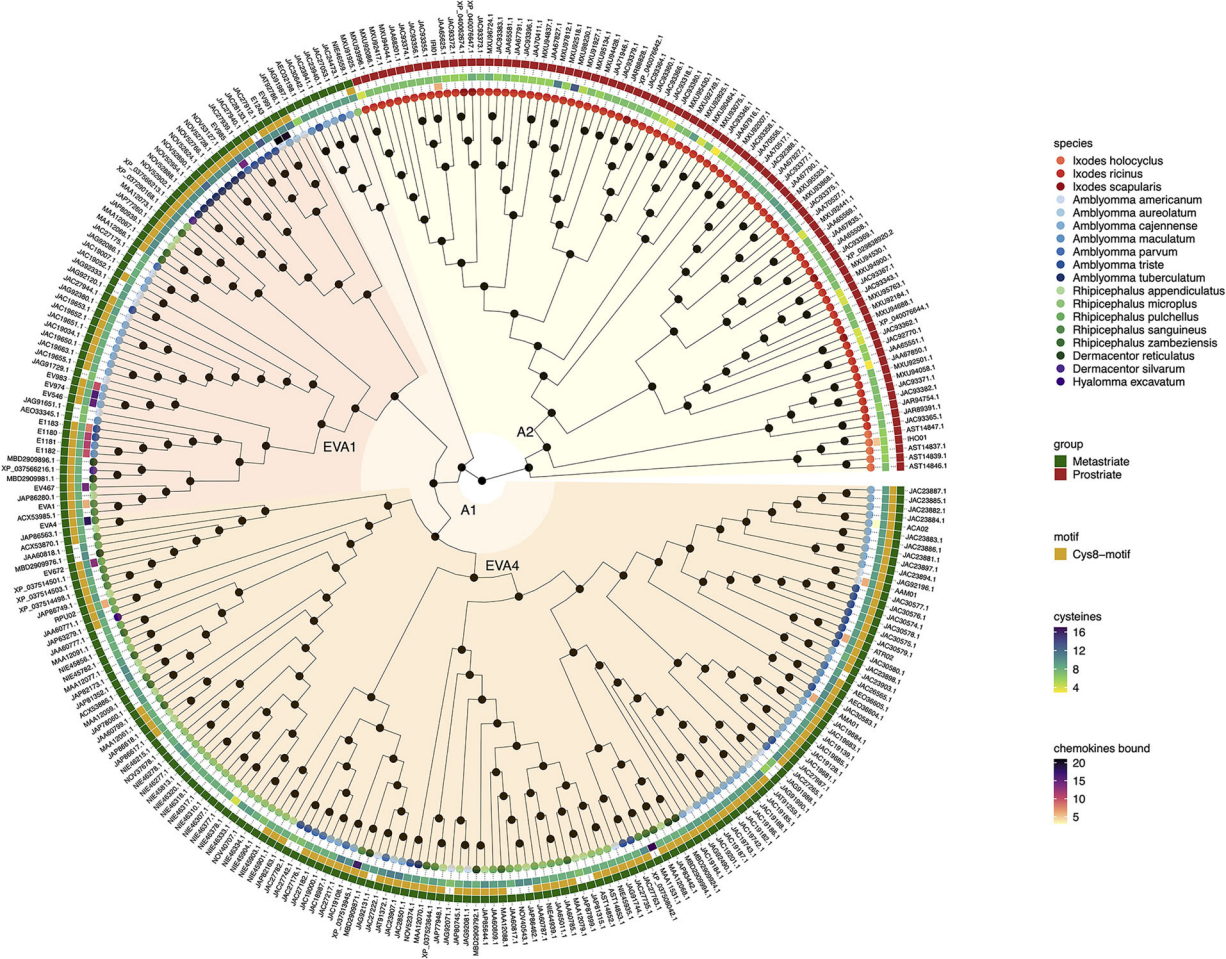
Neighbour-joining tree of class A evasin and evasin-like proteins. Accession numbers of proteins are indicated at the tree tips, which are colour coded by species. The ancestral node at the centre was defined by midpoint rooting. The outermost circle of the heatmap indicates Prostriate or Metastriate lineage. The next two circles of the heatmap indicate the presence of a C-x(14,17) -C-x(3)-C-x(11,16)-C-x(17,20)-C-x(4)-C-x(4,5)-C-x(8)-C (Cys8-motif) and the numbers of cysteines respectively. The innermost circle of the heatmap indicates the number of CC-chemokines bound by biochemically characterized evasins. Classes A1 and A2 were defined as having diverged from the two nodes immediately following the ancestral node and are indicated in orange and yellow respectively. Distinct subclades of class A1 containing EVA1 and EVA4 proteins are indicated in red and dark orange respectively.

**Figure 2:**
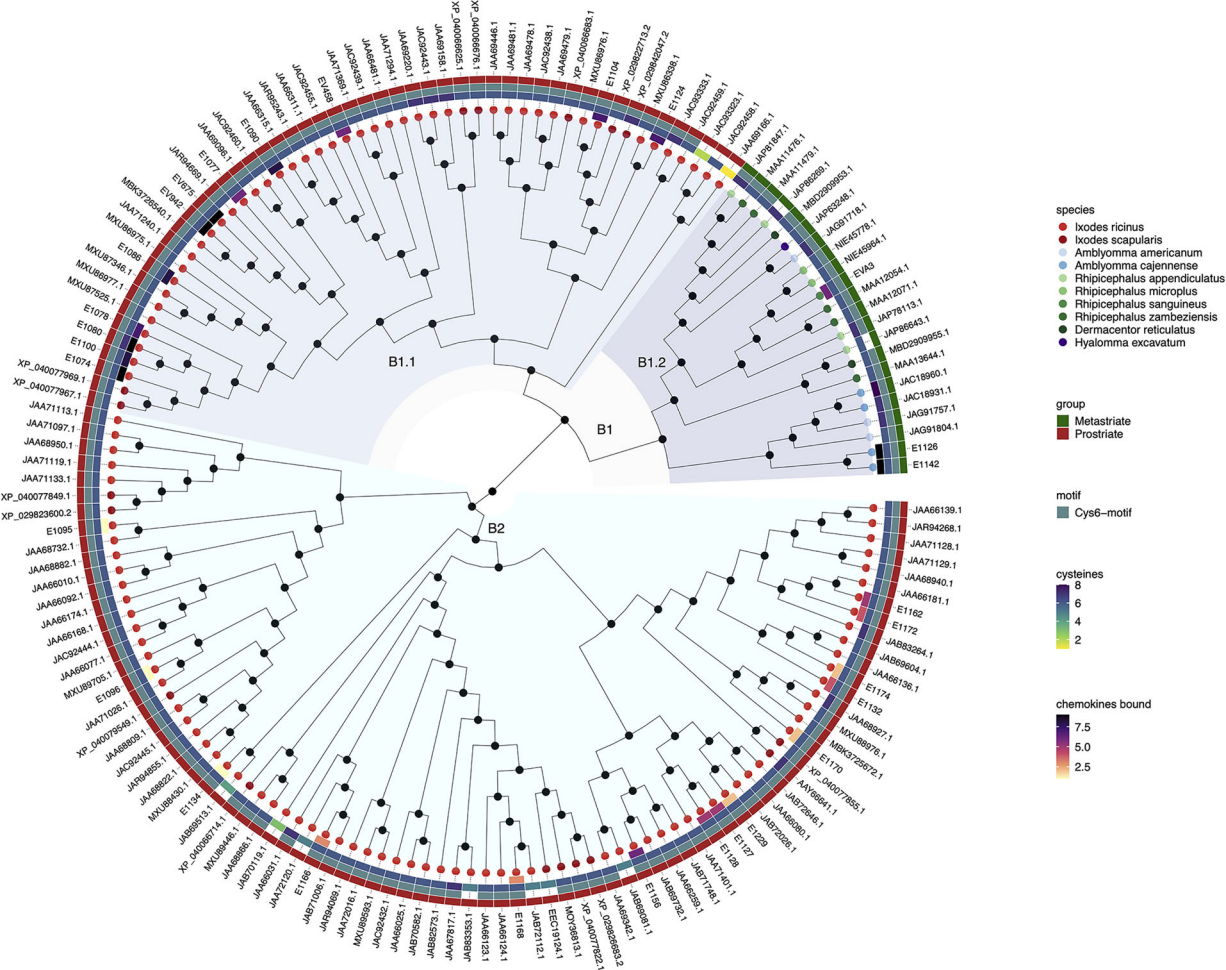
Neighbour-joining tree of class B evasin and evasin-like proteins. Accession numbers of proteins are indicated at the tree tips, which are colour coded by species. The ancestral node at the centre was defined by midpoint rooting. The outer circle of the heatmap indicates Prostriate or Metastriate lineage. The next two circles of the heatmap indicate the presence of a C-x(3)-C-x(6,10)-C-x(3,6)-C-x(1)-C-x(10,11)-C motif (Cys6-motif) and the numbers of cysteines respectively. The innermost circle of the heatmap indicates the number of CXC-chemokines bound by biochemically characterized evasins. Clades B1 and B2 were defined as having diverged from the two nodes immediately following the ancestral node and are indicated in azure and cyan respectively. Subclades B1.1 and B1.2 were defined as having diverged from the two nodes immediately following the node defining clade B1 and are indicated in light and dark azure respectively.

The evolutionary relationships between sequences and groups of sequences can be inferred from phylogenetic trees that are rooted in an explicit ancestral node (Wilkinson et al., 2007). To understand the evolutionary history and relationships between the 292 class A and 157 class B evasin and evasin-like proteins of Prostriate and Metastriate ticks, we performed sequence alignment followed by phylogenetic tree construction using the midpoint rooting method (Hess and De Moraes Russo, 2007; Kinene et al., 2016) to locate the ancestral nodes (**Figures 1**, **2**). We were unable to use the alternative outgroup rooting method as no appropriate outgroup sequence, i.e., an evasin from a species that diverged prior to the separation of Prostriate and Metastriate lineages could be identified.

Inspection of the tree created from class A evasins/evasin-like proteins indicates that the two nodes immediately following the ancestral node clearly separate Prostriate and Metastriate lineages (**Figure 1** and **Supplementary Table S1**). We classified the two clades descending from these nodes as class A1 (Metastriate, 205 members) and class A2 (Prostriate, 87 members) respectively. As shown in the heatmap accompanying the tree, class A1 evasins/evasin-like proteins typically have 8 or more cysteine residues that are appropriately spaced to create a Cys8-motif. Class A2 members typically have less than 8 cysteine residues and lack the Cys8-motif. As also seen in the heatmap, several class A1 evasins have less than the minimal number of Cys residues needed to form the four-disulfide bonded tertiary structure, which is thought to play an important role in chemokine binding (Dias et al., 2009; Denisov et al., 2020).

Inspection of the tree created from class B evasins/evasin-like proteins shows that, unlike the situation with class A evasins, there is incomplete separation between proteins from Prostriate and Metastriate lineages (**Figure 2** and **Supplementary Table S2**). The two nodes immediately following the ancestral node define clades B1 and B2. B1 contains proteins from both Prostriate and Metastriate lineages, whereas B2 is exclusively Prostriate. Within B1 there are two subclades, B1.1 being exclusively Prostriate, and the other, B1.2, exclusively Metastriate. As shown in the heatmap, class B evasin-like proteins from B1.1, B1.2 and B2 clades typically have 6 cysteine residues appropriately spaced to create the Cys6-motif, and can be considered as homologous (**Figure 2** and **Supplementary Table S2**). For subsequent analyses we therefore considered class B proteins as a single class. As also seen in the heatmap, several class B evasins have less than the minimal number of Cys residues needed to form the three-disulfide bonded tertiary structure, which plays an important role in chemokine binding (Lee et al., 2019).

**Figure 3:**
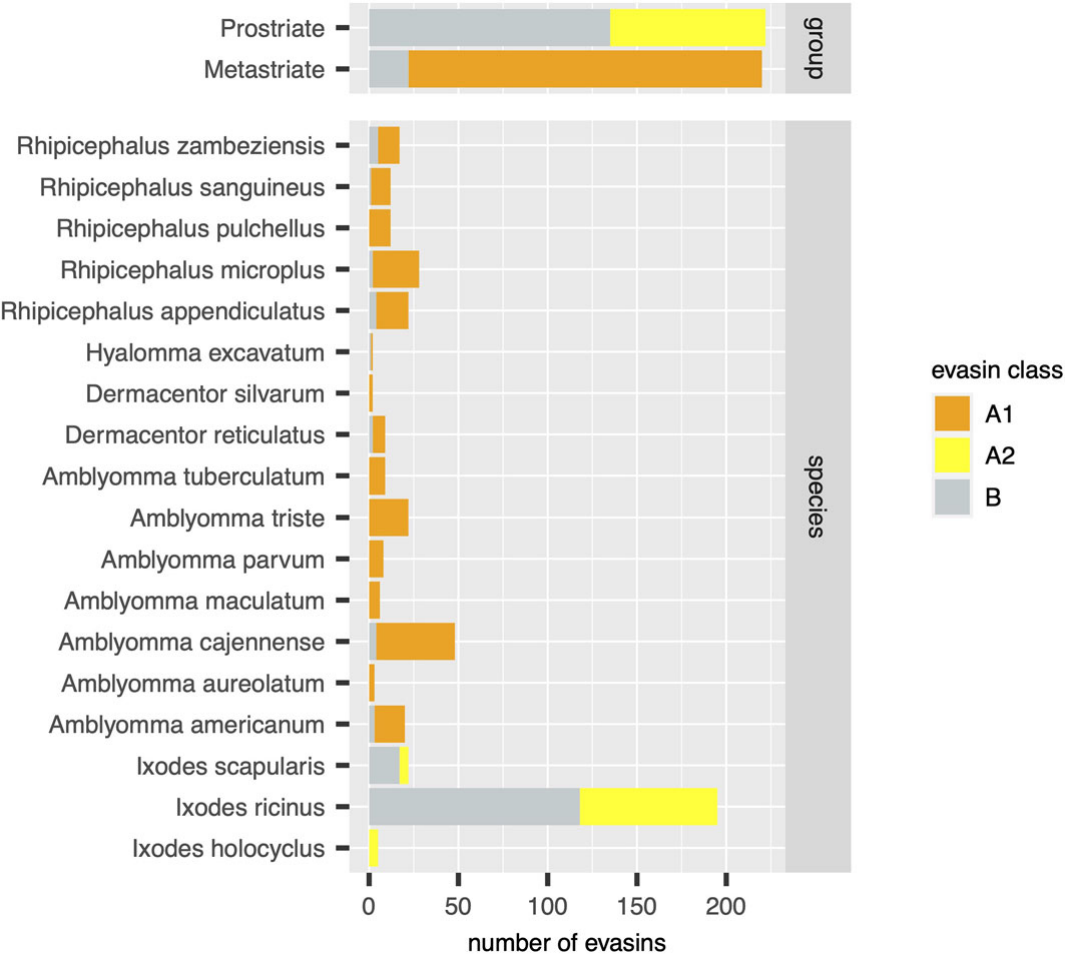
Distribution of evasins and evasin-like proteins in diverse tick species. Stacked bar charts showing the numbers of distinct evasins by class (x-axis, coloured as indicated in the guide) in tick groups (top panel) and in individual tick species (bottom panel).

**Figure 4:**
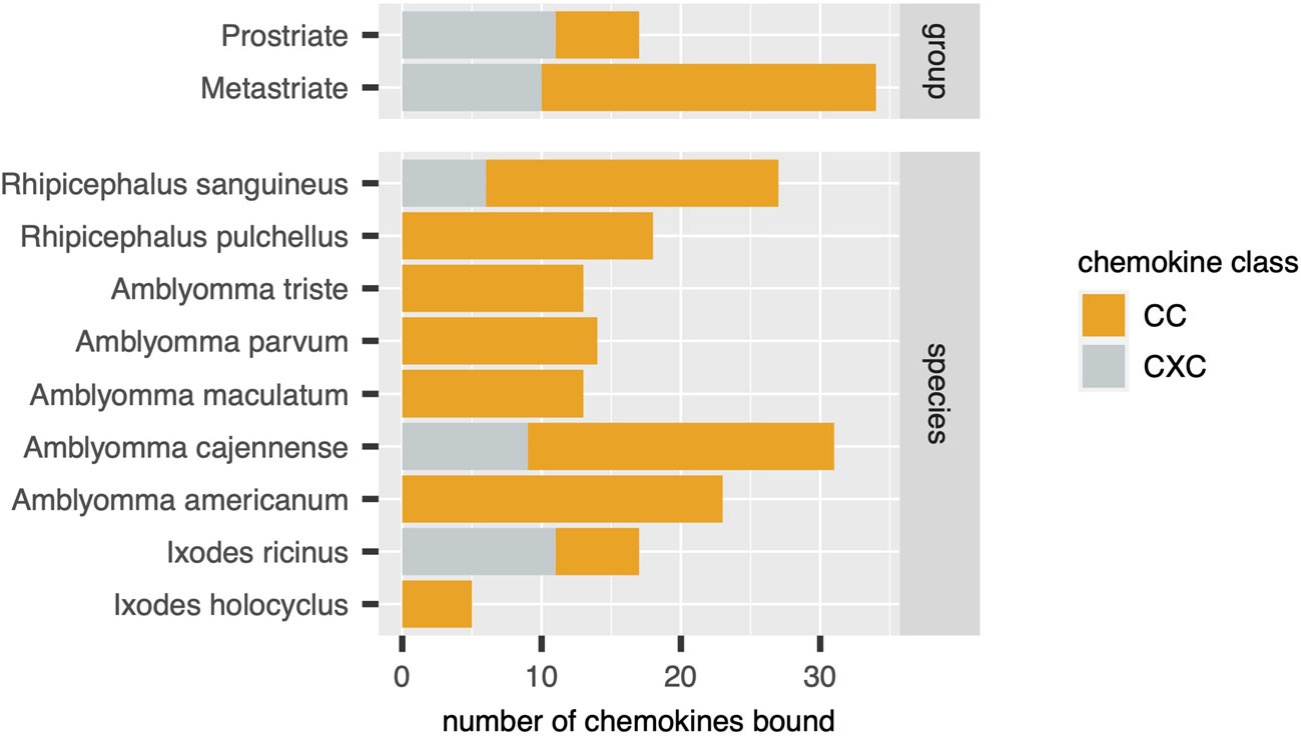
Distribution of chemokines bound by biochemically characterized evasins in diverse tick species. Stacked bar charts showing the numbers of distinct chemokines by class (x-axis, coloured as indicated in the guide) in tick groups (top panel) and in individual tick species (bottom panel).

Using the output of the above analyses, we next estimated the relative numbers of class A1, A2 and B evasins/evasin-like sequences in Prostriate and Metastriate lineages, and in the species constituting these lineages (**Figure 3**). Class B evasins/evasin-like sequences are identified in two Prostriate species (*Ixodes ricinus*, and *Ixodes scapularis*) and four Metastriate genera (*Rhipicephalus, Hyalomma, Dermacentor* and *Amblyomma).* Class A1 evasins/evasin-like sequences are identified in four Metastriate genera (*Rhipicephalus, Hyalomma, Dermacentor* and *Amblyomma).* Class A2 evasins/evasin-like sequences are identified in three Prostriate species (*Ixodes holocyclus*, *I. ricinus*, and *I. scapularis*). The majority of evasins/evasin-like proteins in Prostriate lineages is class B, whereas in Metastriate lineages it is class A1. In keeping with this distinction, the majority of Metastriate evasins characterised to date neutralize CC-chemokines, whereas the majority of Prostriate evasins neutralize CXC-chemokines (**Figure 4**).

## Discussion

In this study we have used systematic BLAST searches with uniform selection criteria to identify evasin-like proteins from publicly available transcriptome databases. Comparison with annotations in the NCBI databases (**Supplementary Figure S1B**) reveal several differences to our analysis. For instance, our analysis indicates there are 195 evasin/evasin-like proteins for *I. ricinus* and 5 evasin/evasin-like proteins for *I. holocyclus* (**Figure 3**). In contrast, the annotations in the NCBI databases suggest that there are 69 for *I. ricinus* and 12 evasin/evasin-like proteins for *I. holocyclus.* Similarly, of the Metastriate tick sequences our analysis indicates that 48 evasin/evasin-like proteins are distinguishable for *A. cajennense* and three for *A. aureolatum* (**Figure 3**), whereas annotations in the NCBI databases suggest that there are 37 and 11 respectively (**Supplementary Figure S1B**). The differences with NCBI annotations likely reflect the application of our search criteria and differences in analytical methodology. Specifically, we performed searches using all 50 biochemically characterised evasins and restricted the output to proteins that had a BLAST e-value <0.0001, contained a signal peptide, and had no stop codons within the predicted mature protein sequence.

The differences between species may also arise partly from differences in the transcript count per species in the NCBI database (**Supplementary Figure S1A**). Moreover, composition of the salivary gland transcriptome varies according to the origin of the tick (geographical location, laboratory colony, etc.), developmental stage and gender, whether from fed, feeding, or unfed ticks, the time of feeding relative to attachment, and the host on which the tick is feeding (De Castro et al., 2016; Garcia et al., 2020; Kim et al., 2020). None of these variables are standardised for salivary gland transcriptomes hence, together with differences in transcript quality and quantity, it is perhaps not surprising we found an approximately 100-fold range in number of evasin-like proteins between different species. Also of note are differences between evasin-like proteins identified in salivary gland transcriptomes and in saliva proteomes. For example, putative evasins were not recorded in the saliva proteomes of *R. microplus* (Tirloni et al., 2014; Garcia et al., 2020). Their apparent absence may be explained by their relatively low expression levels (typically <10%) (Karim et al., 2011; De Castro et al., 2016; Garcia et al., 2020). However, 12 putative evasins were annotated in the saliva proteome (and 43 in the salivary gland transcriptome) of *A. americanum*, providing supporting evidence that putative evasins are secreted proteins (Kim et al., 2020). Differences in the numbers of reported putative evasins highlight the need to standardise source material and analytical approaches to unlock the wealth of information in tick salivary gland transcriptomes.

Phylogenetic tree construction using evasins/evasin-like proteins showed that class A evasins are separated into two classes A1 and A2 which are exclusive to Metastriate and Prostriate lineages respectively. Classes A1 and A2 are structurally dissimilar, with members of class A1 typically having the capacity of forming a four-disulfide-bonded structure, and members of class A2 typically lacking such capacity. The presence of EVA1 and EVA4 in distinct subclades of A1 is intriguing as both have a common four-disulfide bonded core structure. However, there are subtle structural differences between the two structures. The EVA4 structure is bulkier and interacts with chemokines *via* the N-terminal domain, unlike EVA1 which binds through both N and C-terminal domains (Denisov et al., 2020). It is possible that the two subclades reflect these subtle differences. Supporting this idea, E672, a member of the EVA4 subclade, also interacts with chemokines through the N-terminal domain (Eaton et al., 2018; Darlot et al., 2020). In contrast to class A, class B evasins are present in both Prostriate and Metastriate lineages, and members typically have the capacity to form a three-disulfide-bonded “knottin” structure irrespective of lineage. Notably, several class A1 and class B evasins have less than the minimal number of Cys residues needed to form the appropriate Cys–Cys bonded tertiary structure, which is thought to play an important role in chemokine binding (Dias et al., 2009; Lee et al., 2019). It remains to be determined whether such evasin-like proteins are capable of binding chemokines. If neutral evolution and genetic drift are the major reasons for diversity in salivary gland proteins (Mans et al., 2017), many evasin-like proteins may be sub- or non-functional or have new (non-evasin) functions – a hypothesis that needs to be tested.

Taken together these results suggest that class B evasins, which are identified in four Metastriate genera and two Prostriate species, arose prior to the divergence of Prostriate and Metastriate lineages, which occurred ~200-249 MYA (Mans et al., 2012; Jia et al., 2020), and likely functioned to neutralize CXC-chemokines and support blood feeding. The presence of class A1 in four Metastriate genera suggests that these proteins appeared before Metastriate diversification, which occurred ~138 MYA (Jia et al., 2020). The presence of class A2 in three Prostriate species suggests that these proteins appeared before Prostriate diversification, which occurred ~217 MYA (Mans et al., 2012). Our analysis indicates that classes A1 and A2 are structurally dissimilar and are exclusive to Metastriate and Prostriate ticks respectively. Possible explanations for this exclusivity include: a) descent with modification from a shared ancestral protein sequence present before the divergence of Metastriate and Prostriate lineages, b) descent from independent ancestral protein sequences with loss of a class [i.e. “gene death” (Nei and Rooney, 2005)] following Metastriate/Prostriate divergence, or c) origin by horizontal gene transfer from another organism – a phenomenon that has been shown to occur in arthropod lineages and has functional importance at the tick host interface (Chou et al., 2015; Hayes et al., 2020). These alternative scenarios may be resolved by further analysis of ticks from different lineages.

## Conclusions

Our analysis of publicly available tick transcriptomes indicates that evasins/evasin-like proteins of ixodid ticks fall into three distinct classes. Class A1 is exclusive to Metastriate ticks. Members typically contain a characteristic Cys8-motif that is predicted to form a four-disulfide-bonded structure. Two subclades containing EVA1 and EVA4 are evident and are likely to have subtle structural differences. Characterised members exclusively bind CC-chemokines. Class A2 is exclusive to Prostriate ticks, members do not contain a Cys8-motif, and characterised members bind CC-chemokines. Class B is present in both Prostriate and Metastriate ticks, members typically contain a characteristic Cys6-motif predicted to form a three-disulfide-bonded “knottin” structure, and characterised members exclusively bind CXC-chemokines. Our analysis suggests that class B evasin-like proteins arose before the divergence of Prostriate and Metastriate lineages and likely functioned to neutralize CXC-chemokines and support blood feeding. The origin of evasin classes A1 and A2 is yet unclear but may be resolved by transcriptome sequencing from underrepresented tick species. Together with biochemical characterisation of individual proteins such studies will provide further insights into the biological functions of tick evasins, the development of therapeutics that target the chemokine system, and the phylogenetic relationships of these “endless forms most beautiful” (Darwin, 1859).

## Data Availability Statement

The original contributions presented in the study are included in the article/**Supplementary Material**. Further inquiries can be directed to the corresponding author.

## Author Contributions

SB performed the data analysis. SB and PN wrote the manuscript. All authors contributed to the article and approved the submitted version.

## Funding

SB is supported by British Heart Foundation Chair (CH/09/003/26631), and British Heart Foundation Program Grant (RG/18/1/33351) Awards. The research was supported by the Wellcome Trust Core Award Grant Number 203141/Z/16/Z with funding from the NIHR Oxford BRC.

## Author Disclaimer

The views expressed are those of the author(s) and not necessarily those of the NHS, the NIHR or the Department of Health.

## Conflict of Interest

The authors declare that the research was conducted in the absence of any commercial or financial relationships that could be construed as a potential conflict of interest.

## Publisher’s Note

All claims expressed in this article are solely those of the authors and do not necessarily represent those of their affiliated organizations, or those of the publisher, the editors and the reviewers. Any product that may be evaluated in this article, or claim that may be made by its manufacturer, is not guaranteed or endorsed by the publisher.

## Supplementary Material

Supplementary Figure 1 Analysis of the tsa_nr and nr databases, restricted to ticks. **(A)** Distribution of all sequences by species (y-axis) and transcript count (x-axis). Species with less than 100 transcripts were not included. **(B)** Distribution of sequences annotated as evasin or putative evasin in the definition line, by species (y-axis) and transcript count (x-axis).

Supplementary Table 1 Class A evasins/evasin-like proteins used to construct the tree in **Figure 1**. Table shows the identity, mature protein sequences, full protein sequences, genus, species, cysteine count in the mature protein, and the presence of a Cys8 or Cys6 motif.

Supplementary Table 2 Class B evasins/evasin-like proteins used to construct the tree in **Figure 2**. Table shows the identity, mature protein sequences, full protein sequences, genus, species, cysteine count in the mature protein, and the presence of a Cys8 or Cys6 motif.
