# Supplementary figures and images for: Phylogenetic Analysis Indicates That Evasin-Like Proteins of Ixodid Ticks Fall Into Three Distinct Classes
Source: Front Cell Infect Microbiol. 2021 Oct 22;11:769542. doi: 10.3389/fcimb.2021.769542 (PMC8569228; doi:10.3389/fcimb.2021.769542)

**A**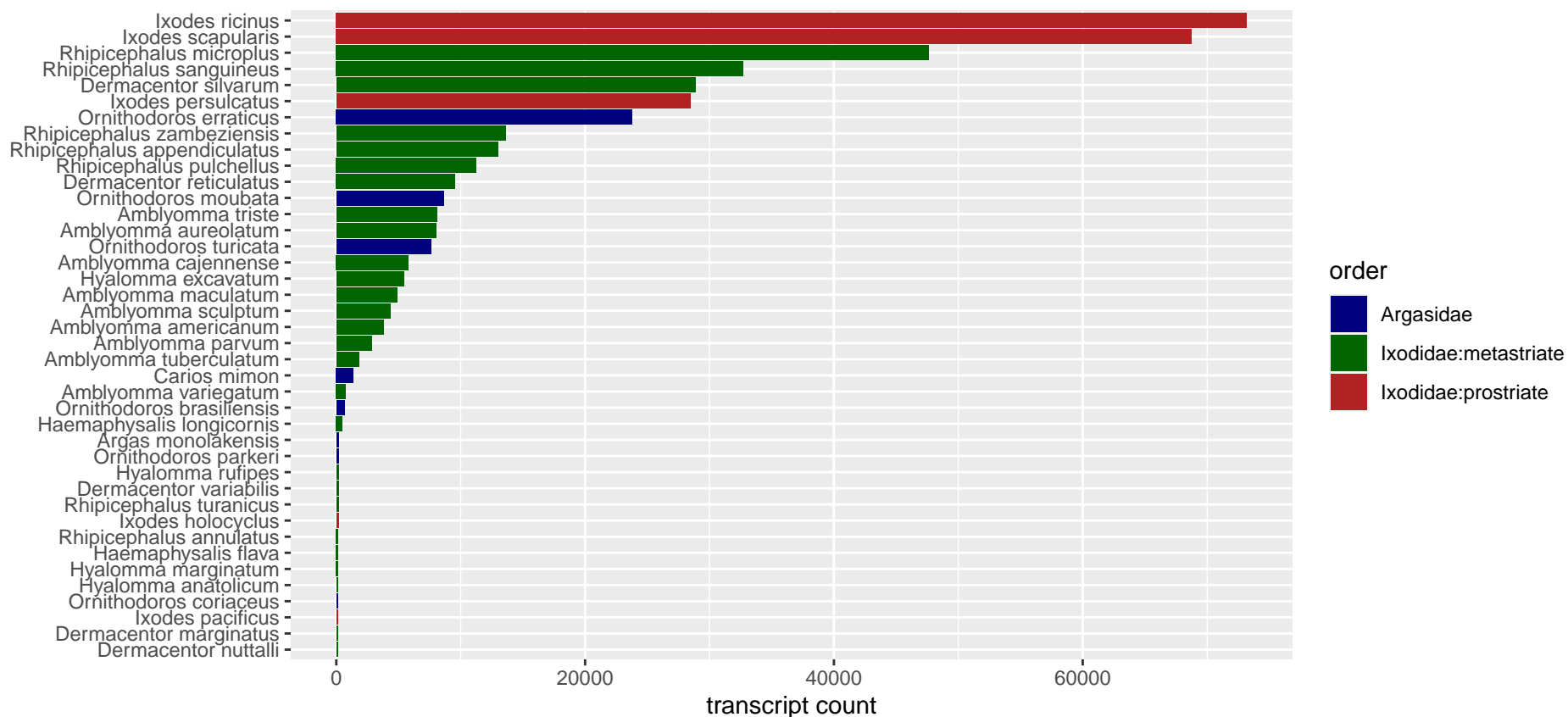**B**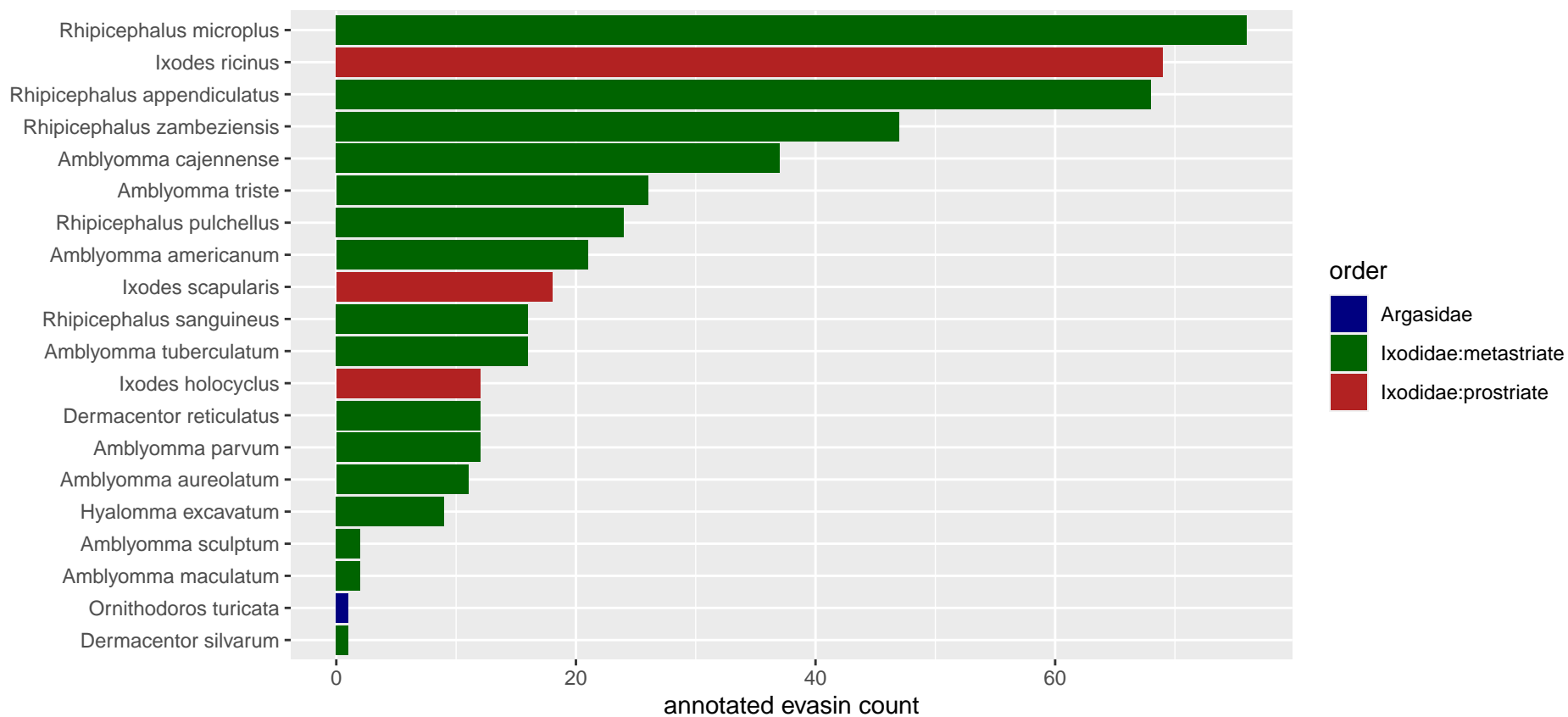

Supplement: Supplementary Figure 1 — Analysis of the tsa_nr and nr databases, restricted to ticks. (A) Distribution of all sequences by species (y-axis) and transcript count (x-axis). Species with less than 100 transcripts were not included. (B) Distribution of sequences annotated as evasin or putative evasin in the definition line, by species (y-axis) and transcript count (x-axis). [file DataSheet_1.pdf]
